# Activation Energy, Temperature Exponent, and Adiabatic Laminar Flame Speed of Preheated Syngas Mixtures from Biomass-Derived Vapors

**DOI:** 10.1021/acsomega.6c01849

**Published:** 2026-04-15

**Authors:** Gabriel Nagafugi de Souza Costa, Roberto Wolf Francisco Jr., Amir Antônio Martins Oliveira

**Affiliations:** † Mechanical Engineering Department, 74382 Santa Catarina State University, Joinville 89219-710, Brazil; ‡ Mechanical Engineering Department, Federal University of Santa Catarina, Florianopolis 88040-900, Brazil

## Abstract

Reforming biomass-derived vapors maximizes the utilization of fuels obtained from biomass gasification and pyrolysis. The gas produced can be added to the feedstock for the synthesis of synthetic fuels and chemicals or directly burned to produce heat and power. Knowledge of the laminar flame speed, activation energy, and temperature exponent enables rapid design and analysis of combustion chambers using one-step global chemical kinetic mechanisms. The objective of this work is to evaluate the effect of unburned mixture temperature on adiabatic laminar flame speed, overall activation energy, and temperature exponent for H_2_/CO/CO_2_/N_2_ mixtures, representative of dry reforming of biomass-derived vapors, with H_2_/CO ratios from 0.43 to 1.0. The reactant temperature ranged from 300 to 400 K, while the equivalence ratio was kept at 0.9. The flat flame with heat loss method was used for the simultaneous measurement of the adiabatic laminar flame speed and overall activation energy. The temperature exponent was determined by two methods. The first uses the power-law relation between the laminar flame speed and unburned mixture temperature. The second estimate is from the measured activation energy. The results from the measurements were compared with calculations using the chemical kinetic mechanisms FFCM-1, HP-Mech, San Diego and Goswami. Adiabatic laminar flame speed increased more strongly with unburned mixture temperature in H_2_-rich mixtures. The activation energy showed little sensitivity to the unburned mixture temperature, especially in mixtures with lower H_2_ concentrations. For a mixture with 15% H_2_, the temperature exponent determined from the activation energy ranged from 2.0 to 2.2 for unburned mixture temperatures between 325 and 500 K, which is consistent with a first-order effective reaction order with respect to the fuel concentration. The adiabatic laminar flame speeds extrapolated using the power-law relation with the temperature exponents from both methods showed greater accuracy for temperature increases of less than 100 K relative to the reference condition, regardless of the H_2_ content.

## Graphical abstract

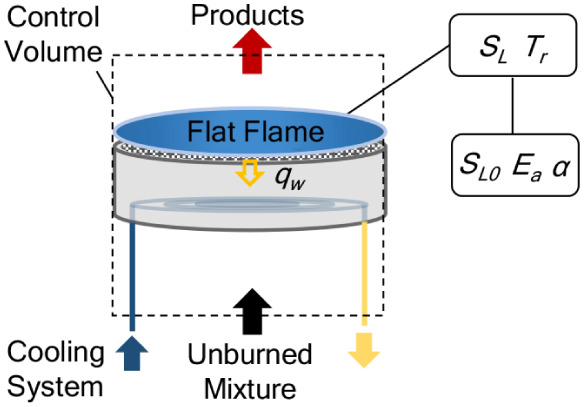

## 1 Introduction

Biomass gasification produces both gaseous and condensable species. The noncondensable fraction, commonly referred to as synthesis gas (syngas), is widely used for the production of synthetic fuels and industrial chemicals, such as methanol and ammonia, as well as for heat and power generation. The condensable vapors can be further reformed to enhance the overall utilization of biomass gasification and pyrolysis products.^1^ The resulting gas from reforming may either be reintegrated into the feedstock for the synthesis of fuels and chemicals or directly combusted for heat and power generation.^1^

The products obtained from reforming, typically composed of H_2_, CO, CO_2_, N_2_, and CH_4_, can exhibit significant variability in composition.^2^ Such variations strongly affect the adiabatic laminar flame speed (*S*_L0_), which is a key parameter in the design and optimization of combustion systems. In addition to composition, *S*_L0_ is influenced by the equivalence ratio, as well as the pressure and temperature of the unburned mixture.^3^ Furthermore, knowledge of the laminar flame speed, activation energy, and temperature exponent enables the development of one-step global chemical kinetic mechanisms, which are essential for fast analytical and numerical modeling of combustion devices.

Several studies have investigated the effects of the composition and operating parameters on the adiabatic laminar flame speed of syngas. The influence of CO_2_ and N_2_ dilution in H_2_-rich mixtures (90% H_2_ and 10% CO) shows that CO_2_ dilution leads to a greater reduction in flame speed compared to N_2_.^4^ This behavior is also observed for mixtures containing 50% H_2_ and 50% CO.^5^ Further analysis has been performed by independently varying the CO_2_/N_2_ and H_2_/CO ratios, allowing the separation of physical and chemical effects and providing deeper insight into the role of mixture composition on flame propagation.^6^ In addition, increasing CH_4_ content (up to 20% vol.) in syngas mixtures leads to a reduction in *S*_L0_, primarily due to the decrease in H_2_ concentration, which has a more pronounced influence than changes in the diluent composition.^7^

However, most studies available in the literature have been conducted using simplified mixtures with only two or three components and under ambient unburned mixture temperature conditions.^8–10^ Only a limited number of works have investigated multicomponent syngas mixtures under elevated temperature and pressure conditions,^11,12^ despite the fact that many practical applications operate far from ambient conditions.

The traditional relation expressing the dependence of the adiabatic laminar flame speed on the unburned mixture temperature is the power-law relation^13^

1 $$\left(\frac{S_{L0}}{S_{L0,\mathrm{ref}}}\right)={\left(\frac{T_{u}}{T_{u,\mathrm{ref}}}\right)}^{\alpha_{T}}$$

This relationship can be used to determine the value of the temperature exponent (α_T_) by measuring the adiabatic laminar flame speed (*S*_L0_) for different unburned mixture temperature (*T*_u_). The values of α_T_ obtained are averages in the temperature range covered by the data.

Then, knowing α_T_ and selecting measured values of the adiabatic laminar flame speed and unburned mixture temperature to represent the reference condition (*S*_L0,ref_ and *T*_u,ref_), *S*_L0_ can be continuously calculated as a function of *T*_u_. This is the most traditional method and has been used by many authors.^14–16^ The accuracy of the values of *S*_L0_ obtained using the power-law relation depends on the precision of the measured values of *S*_L0,ref_ and α_T_. The extrapolation of *T*_u_ to values outside the measured range increases the associated errors in the estimation of *S*_L0_.

Another method for estimating the temperature exponent at different unburned mixture temperatures was presented by Han et al.^17^ The authors considered two flames differing only by the unburned mixture temperature *T*_u_ and developed a relation between the temperature exponent (α_Ea_) and the overall activation energy (*E*_a_) in the form

2 $$\alpha_{\mathrm{Ea}}=\frac{E_{a}}{2R}\cdot \frac{1}{\ln\left(T_{u}\right)-\ln\left(T_{u,\mathrm{ref}}\right)}\cdot \left(\frac{1}{T_{\mathrm{ad},\mathrm{ref}}}-\frac{1}{T_{\mathrm{ad}}}\right)+1$$

In eq 2, *T*_u,ref_ and *T*_ad,ref_ are the unburned mixture temperature and the adiabatic flame temperature at the reference condition, respectively, and *R* is the universal gas constant. In this model, *E*_a_ is an average value in the temperature range covered by the data.

From this relation, in principle, α_Ea_ can be determined solely from the knowledge of *E*_a_. Alternatively, using the power law relation in eq 1, a value of α_Ea_ is determined for each *T*_u_ and *S*_L0_. Then, from eq 2, a linear regression is applied to a set of points of *T*_u_ and α_Ea_, and an average *E*_a_ over the range of *T*_u_ is obtained. Although α_Ea_ is derived using a different approach than α_T_, both temperature exponents can be employed in conjunction with the power-law relation to estimate the adiabatic laminar flame speed over a range of unburned mixture temperatures, as reported by Han et al.^17^

The activation energy can also be determined using the method presented by ref 18, in which the overall activation energy is given by

3 $$E_{a}=-2R\left\{\frac{\partial [\ln\left(\rho_{g0}S_{L0}\right]}{\partial \left[1/T_{\mathrm{ad}}\right]}\right\}$$

where ρ_g0_ is the specific mass of the reactant mixture.

In this method, the adiabatic laminar flame speed *S*_L0_ is measured for mixtures with different adiabatic flame temperatures *T*_ad_. The flame temperature is changed by the dilution of the reactant mixture using an inert gas. The activation energy obtained from eq 3 is assumed to be constant over the dilution range.

In general, *E*_a_ depends on the composition of the fuel, the equivalence ratio, and the temperature and pressure of the reactant mixture.^19^ The effect of the syngas mixture composition on *E*_a_ was observed in a previous work by Costa et al.,^20^ for equivalence ratios between 0.8 and 1.0 and a wide range of N_2_/CO_2_ dilution. However, the relationship between the *E*_a_ values of syngas mixtures for different unburned mixture temperatures has not yet been studied. A brief literature review presented in the same work^20^ indicates that the determination of the temperature exponent for multicomponent syngas mixtures with different H_2_/CO ratios has also been little explored, and its systematic variation as a function of unburned mixture temperature has not yet been presented.

Thus, the present work aims to evaluate the influence of preheating syngas mixtures on adiabatic laminar flame speed, activation energy, and temperature exponent. The mixtures evaluated are composed of H_2_, CO, CO_2_ and N_2_, with H_2_/CO varying between 0.43 and 1.0, which are typical of dry reforming of biomass-derived vapors from air-steam gasification.^1^

Accordingly, this study provides the following contributions: (1) an evaluation of the sensitivity of the overall activation energy of multicomponent syngas mixtures to variations in unburned mixture temperature under fixed equivalence ratio, pressure, and composition; (2) a systematic characterization of the temperature exponent for multicomponent syngas as a function of both reactant temperature and H_2_/CO concentration; (3) an evaluation of the effect of the H_2_/CO ratio in low-hydrogen syngas mixtures (H_2_ <15%) on the adiabatic laminar flame speed; and (4) an analysis of the predictive performance of different chemical kinetic mechanisms for the *S*_L0_ of preheated syngas mixtures with different H_2_/CO ratios.

## 2 Experimental and Numerical Procedures

The method selected to measure the adiabatic laminar flame speed was the flat flame method with heat loss.^19,21^ The advantage of this method is the possibility of measuring the adiabatic laminar flame speed and determining the apparent activation energy from the same data set obtained in a single run, for the same unburned mixture temperature and equivalence ratio, thus minimizing error propagation.^22^ In this work, the temperature exponent is determined by two different methods, the power-law relation, eq 1, and the *E*_a_ method through eq 2 presented by Han et al.^17^ The measurements are compared to predictions using the chemical kinetic mechanisms FFCM-1,^23^ HP-Mech,^24^ San Diego,^25^ and Goswami,^26^ using the PREMIX code of ChemKin PRO, and the discrepancies are analyzed.

This work extends the previous work by Costa et al.,^20^ which evaluated the effects of the composition and dilution of syngas mixtures on adiabatic laminar flame speed. Table 1 shows the syngas compositions and unburned mixture temperatures used in this work. All measurements were carried out at 0.1 MPa and a fuel equivalence ratio of 0.9.

**Table 1:** Fuel Mixtures and Test Conditions of the Present Work (Ø = 0.9 and *P* = 0.1 MPa)

	Syngas composition (% vol.)						
Fuel mixtures	H_2_	CO	CO_2_	N_2_	H_2_/CO	*T*_u_ [K]	LHV [kJ/kg]
S1	15	15	15	55	1.0	300–350–400	2969
S2	12	18	15	55	0.67	300–350–400	2930
S3	9	21	15	55	0.43	300–350–400	2892

The experimental setup and procedures applied in the present work are basically the same presented by Costa et al.^20^ In reference 20, the measurements of adiabatic laminar flame speed of methane–air mixtures at 298 K obtained from the experimental setup used in the present work were compared with the values presented by others over the last 20 years, and the activation energy was compared with experimental and numerical results. Deviations smaller than 1.5 cm/s were found, and therefore, the experimental setup and procedure were considered validated.

The main difference in the experimental setup used in this work with that of Costa et al.^20^ is the use of a preheater to raise the air temperature before mixing with the fuel. The electrical preheater is composed of a central tube surrounded by electrical resistances, controlled by a PID microprocessor connected to an RTD PT100 (class A 1/10). This RTD was positioned at the burner inlet and was used to measure and control the unburned mixture temperature.

Table 2 presents the chemical kinetic mechanisms used in this work. The adiabatic laminar flame speed was obtained numerically using the PREMIX code of ChemKin PRO, with the GRAD and CURV parameters set to 0.01 to improve convergence, as presented by ref 20.

**Table 2:** Chemical Kinetic Mechanisms Were Selected

Mechanism	Version	Reference
FFCM-1	2016	^23^
San Diego	2016	^25^
HP-Mech	2017	^24^
Goswami	2014	^26^
GRI-Mech	3.0	^27^

The numerical analysis of the effect of reactant preheating on the adiabatic laminar flame speed was performed by increasing the unburned mixture temperature.

## 3 Results and Discussion

### 3.1 Analysis of the Temperature Exponent for Methane–Air Mixtures

The objective of this section is to verify whether the power-law relation, eq 1, can be used to predict the adiabatic laminar flame speeds of methane–air mixtures using the values of the temperature exponent obtained from the method by Han et al.,^17^ eq 2, but using the value of *E*_a_ obtained for a constant temperature of 298 K. Table 3 summarizes the measurements of adiabatic laminar flame speed and overall activation energy from ref 20 at equivalence ratios of 0.8, 0.9, and 1.0, *T*_u_ = 298 K, and *P* = 0.1 MPa.

**Table 3:** Adiabatic Laminar Flame Speed and Overall Activation Energy Obtained for Methane–Air Mixtures Using the Experimental Setup of the Present Work, for Ø between 0.8 and 1.0, *T*_u_ = 298 K, and *P* = 0.1 MPa (Reproduced from ref 20)

Ø	*S*_L0_ (cm/s)	*E*_*a*_ (kJ/mol)
0.8	27.9	319
0.9	31.6	184
1.0	36.3	231

The *E*_a_ results reported in Table 3 were used to determine α_Ea_ from eq 2 for reactant temperatures ranging from 300 to 450 K, assuming that the activation energy determined at 298 K remains constant. Figure 1 presents the temperature exponent determined from eq 2 using the *E*_a_ values presented in Table 3, as a function of the unburned mixture temperature (325 to 600 K) for premixed methane and air mixtures, at *P* = 0.1 MPa and Ø between 0.8 and 1.0.

**Figure 1:**
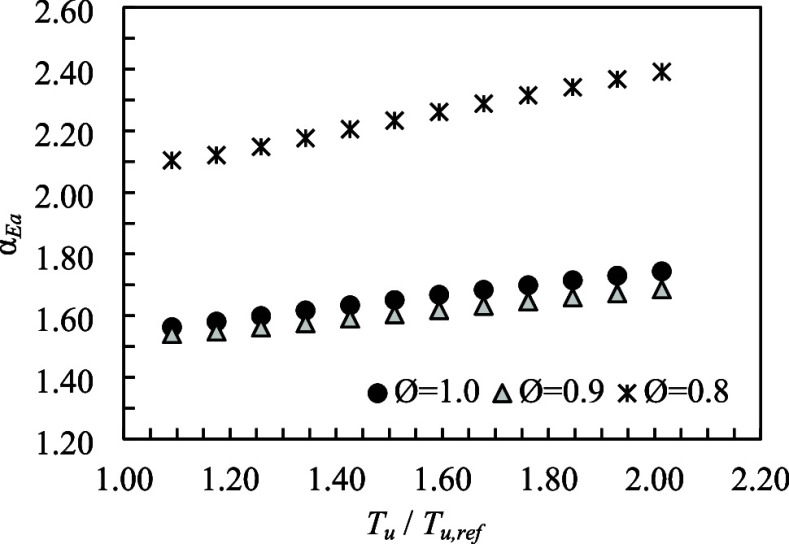
Temperature exponent as a function of the preheating unburned mixture temperature for methane–air mixtures at *P* = 0.1 MPa and *T*_u,ref_ = 298 K.

The temperature exponent increases with increasing unburned mixture temperature, a trend that has also been observed both numerically and experimentally.^17^ For unburned mixture temperatures between 298 and 368 K, using the GRI-Mech 3.0 mechanism, values between 1.57 and 1.62 were reported for Ø = 1.0 at *P* = 0.1 MPa.^17^ In the present work, α_Ea_ varies between 1.56 and 1.60 for unburned mixture temperatures ranging from 325 to 375 K.

A decrease in the equivalence ratio leads to an increase in the temperature exponent. For Ø = 0.8, α_Ea_ ranges from 2.1 to 2.3 over a temperature interval of 325 to 600 K, corresponding to values approximately 16% higher than those reported in ref 17. For Ø = 0.9, the α_Ea_ values are slightly lower than those for Ø = 1.0, by approximately 2–3%.

Using the traditional method to determine the temperature exponent (α_T_) for a stoichiometric methane–air mixture, a value of 1.82 was reported in ref 28, while for Ø = 0.8, α_T_ increases to 2.06. It is important to note that these values represent averages over a temperature range between 350 and 650 K, whereas in the present work, α_Ea_ was determined individually for each unburned mixture temperature (*T*_u_).

Gu et al.,^29^ applying the same traditional method of Varghese et al.,^28^ obtained α_T_ equal to 1.612 and 2.105 for Ø equal to 1.0 and 0.8, respectively. In general, the temperature exponent can vary around 1.55 and 2.0 for stoichiometric conditions and between 1.8 and 2.3 for Ø equal to 0.8.^13^

Now, from the values of α_Ea_ determined at each *T*_u_ (Figure 1), the adiabatic laminar flame speed was estimated using the power-law relation, eq 1. Figure 2 presents the adiabatic laminar flame speed of methane–air mixtures as a function of unburned mixture temperature for *P* = 0.1 MPa and Ø between 0.8 and 1.0. The black curves (solid and dotted lines) represent the laminar flame speeds *S*_L0_ calculated by using the GRI-Mech 3.0 and San Diego (2016) chemical kinetic mechanisms. The red dashed-dotted line corresponds to the extrapolated adiabatic laminar flame speed, obtained using the temperature exponent determined in the present study (see Figure 1). The error bars represent the uncertainty associated with the extrapolated flame speed, estimated through error propagation analysis. The results were also compared with experimental values presented by others.^29–32^

**Figure 2:**
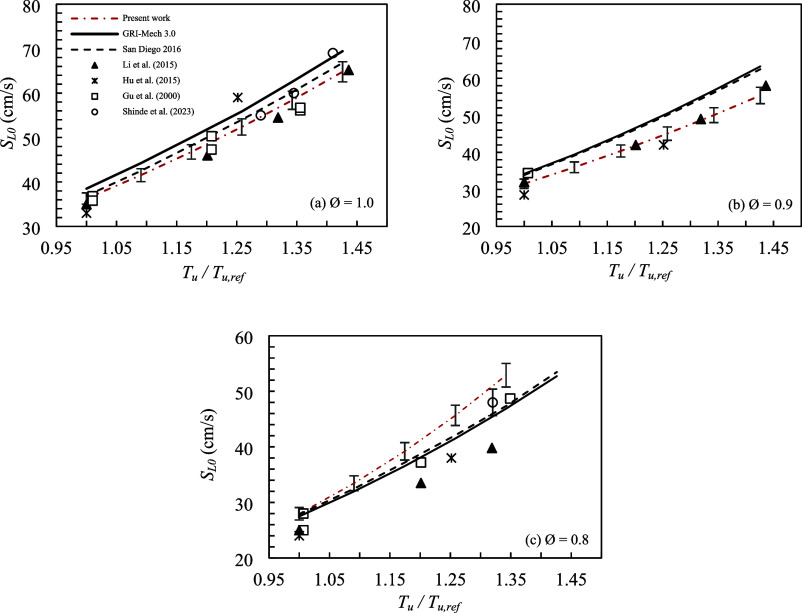
Adiabatic laminar flame speed for methane–air mixtures as a function of unburned mixture temperature for Ø of (a) 1.0, (b) 0.9, and (c) 0.8.

At the stoichiometric condition (Figure 2a), the differences between the measurements in the present work and the numerical predictions varied from 6 to 7% for GRI-Mech 3.0 and from 1 to 3% for San Diego (2016), for the unburned mixture temperatures between 298 and 425 K. At Ø = 0.9, the differences between the measurements and the numerical predictions ranged from 8 to 12% for GRI-Mech 3.0 and from 7 to 13% for San Diego (2016), for *T*_u_/*T*_u,ref_ between 1.00 and 1.43. However, the adiabatic laminar flame speed determined experimentally for *T*_u_ = 298 K presented values 8% and 7% lower than GRI-Mech 3.0 and San Diego (2016), respectively. Thus, it can be observed that the difference obtained for *S*_L0_ at the reference temperature (298 K) propagated across the entire temperature range analyzed. Furthermore, the results obtained by other authors for Ø = 0.9 presented values lower than those predicted by the chemical kinetic mechanisms, such as Hu et al.^30^ or Li et al.,^31^ with differences around 8% for *T*_u_/*T*_u,ref_ equal to 1.44. It is also noted that the results obtained in this work showed the same trend as the values measured by other authors.

For Ø = 0.8, the percentage differences between the adiabatic laminar flame speeds obtained in the present work and numerical predictions range from 1% to 15% (up to 5 cm/s) over the temperature interval from 300 to 400 K. At 402 K (*T*_u_/*T*_u,ref_ = 1.35), an adiabatic laminar flame speed of approximately 48.7 cm/s was reported in ref 29, corresponding to a difference of about 7% (4 cm/s) relative to the present results. Furthermore, in a more recent study conducted at 393 K (*T*_u_/*T*_u,ref_ = 1.32), the difference between the present results and those reported in ref 32 is approximately 6% (around 3 cm/s), which is comparable to the reported experimental uncertainty of 5%.

In contrast to the behavior observed for equivalence ratios of 1.0 and 0.9, the condition at Ø = 0.8 still shows noticeable discrepancies among the adiabatic laminar flame speed values reported in the literature. For *T*_u_/*T*_u,ref_ = 1.32, the difference between the values reported in refs 32 and 31 is approximately 8 cm/s, highlighting the experimental variability associated with leaner mixtures.

Comparisons with the numerical predictions using GRI-Mech 3.0 and San Diego (2016) showed greater differences at high temperatures and equivalence ratios below stoichiometry. However, the divergence of the numerical results when compared with experimental values for the methane and air mixture using different chemical kinetic mechanisms has already been reported.^3^

### 3.2 Effect of Reactant Preheating and Syngas Composition on *S*_L0_ and *E*_a_

In this section, the effect of reactant preheating on adiabatic laminar flame speed and activation energy was evaluated for mixtures S1, S2, and S3. The unburned mixture temperatures used were 298, 350, and 400 K. The equivalence ratio was kept constant at Ø = 0.9 for all measurements.

Figure 3 shows the experimental and numerical results of the adiabatic laminar flame speed as a function of the unburned mixture temperature for mixtures S1, S2, and S3. Numerical simulations were performed using the chemical kinetics mechanisms HP-Mech (2017), San Diego (2016), FFCM-I (2016), and Goswami (2014).

**Figure 3:**
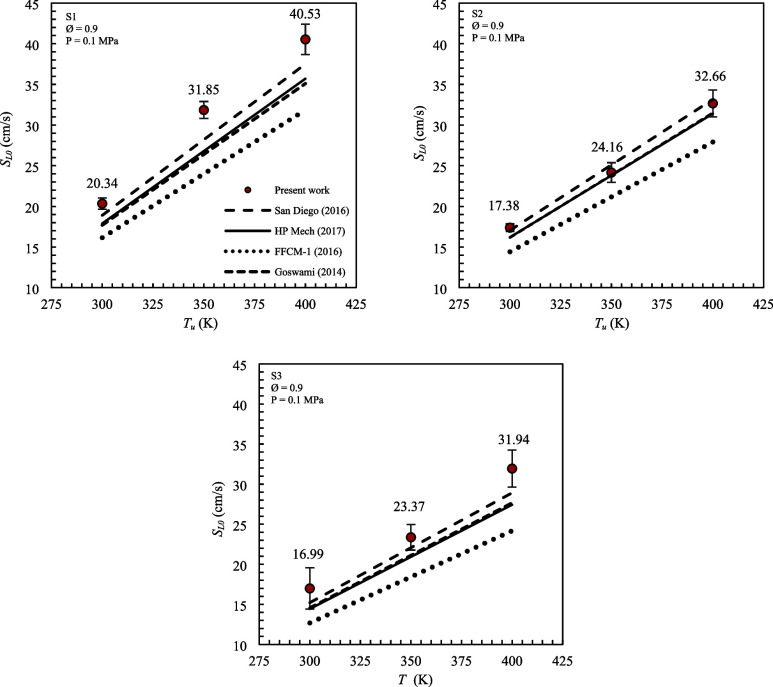
Adiabatic laminar flame speed as a function of unburned mixture temperature for mixtures S1, S2, and S3, with *P* = 0.1 MPa, and Ø = 0.9.

It can be seen that the reactant preheating caused a significant increase in the adiabatic laminar flame speed results. The growth of the flame speed in relation to the initial temperature was verified in all cases evaluated. For the S1 mixture, the increase of the unburned mixture temperature from 298 to 350 K caused an increase of 56.6% in the *S*_L0_, while the S2 and S3 mixtures registered increases of 40% and 37.5%, respectively. For *T*_u_ = 400 K, mixtures S1, S2, and S3 registered adiabatic laminar flame speeds of 40.5, 33.5, and 31.9 cm/s, respectively. The effect of increasing the temperature on the adiabatic laminar flame speed was more significant for mixture S1, which has a higher concentration of H_2_.

Comparing the experimental and numerical results, it can be observed that the mechanism that presented the greatest agreement with the experiment was San Diego (2016), with a maximum percentage difference in relation to the experimental results of 13% for mixture S1 and a minimum of 2% for mixture S2. For mixture S2, differences of 0.35%, 1%, and 2.54% were obtained at temperatures of 298, 350, and 400 K, respectively.

The FFCM-1 (2016) mechanism presented greater differences in relation to the experiment, varying between 17% for mixture S2 and 25% for mixture S3. It is also observed that increasing the unburned mixture temperature led to an increase in the percentage differences for all mixtures evaluated.

Goswami (2014) and HP-Mech (2017) presented very similar results, including increasing the unburned mixture temperature. The experimental adiabatic laminar flame speeds for mixture S2, containing 12% H_2_, resulted in the greatest agreement with the numerical values obtained using these mechanisms.

Figure 4 shows the effect of reactant preheating on the overall activation energy for mixtures S1, S2, and S3. All tests were performed for Ø = 0.9 and *P* = 0.1 MPa.

**Figure 4:**
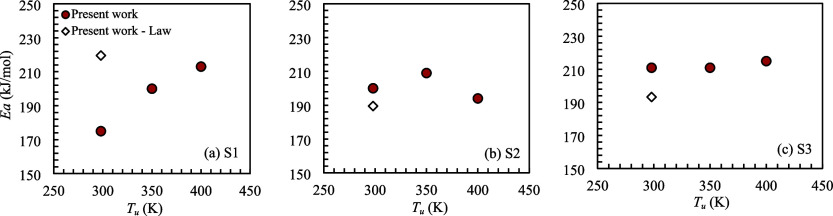
Activation energy as a function of unburned mixture temperature for mixtures (a) S1, (b) S2, and (c) S3. All tests were performed for Ø = 0.9 and *P* = 0.1 MPa.

The methodology presented by Egolfopoulos and Law^18^ was also used to determine the *E*_a_ with the experimental results of *S*_L0_ obtained in the present work (unfilled diamond) for mixtures S1, S2, and S3. However, in this case, it was only possible to obtain a single *E*_a_ for each fuel mixture, which thus represents an average activation energy for the reactant over the temperature range of 298 to 400 K. For mixture S1, the percentage difference of the *E*_a_ determined using the present method and presented by Egolfopoulos and Law^18^ was 25%. For mixtures S2 and S3, the differences were 6 and 8%, respectively.

The measurement uncertainty of *E*_a_ for most of the measured points was on the order of 10%. The activation energy of mixture S1, which has the highest H_2_ content (15% H_2_), showed an increase of approximately 17% when increasing the unburned mixture temperature, ranging from 175 to 213 kJ/mol. For mixtures S2 and S3, with concentrations of 12 and 9% H_2_, respectively, the value of *E*_a_ remained within the measurement uncertainty, on the order of 200 kJ/mol for mixture S2 and 211 kJ/mol for S3.

Regarding mixture S1, the higher H_2_ concentration increases the net reaction rates of H_2_ + OH → H_2_O + H (*E*_a_ = 14.35 kJ/mol) and H_2_ + O → OH + H (*E*_a_ = 26.19 kJ/mol), which dominate the production of H and OH radicals and strengthen the chain branching pathways that control flame propagation, as discussed in ref 20. As the unburned mixture temperature increases, these reactions become even more important, further enhancing the radical pool and increasing *S*_L0_, as shown in Figure 3(a). At higher unburned mixture temperatures, however, other reaction pathways with higher activation energy become active, such as the HO_2_ production from H_2_ + O_2_ → HO_2_ + H (*E*_a_ = 224 kJ/mol), thus increasing the overall activation energy, as shown in Figure 4(a).

### 3.3 Effect of Reactant Preheating and Syngas Composition on the Temperature Exponent

In the present study, the temperature exponent was determined by using two different methods. First, a traditional method was employed, which has been widely used.^29,33^ In this method, the reactants are preheated to different temperatures, and the adiabatic laminar flame speeds are measured. Then, by applying the power-law relation (eq 1) to the set of measurements, the temperature exponent (α_T_) is determined. In this way, the temperature exponent represents an average for a range of measured adiabatic laminar flame speeds at different unburned mixture temperatures. After the temperature exponent is determined, the power-law relation is applied again to estimate the adiabatic laminar flame speed for other desired unburned mixture temperatures.

Table 4 presents the temperature exponents (α_T,exp_) obtained using the measured adiabatic laminar flame speeds for mixtures S1, S2, and S3. The results were compared with the temperature exponents obtained numerically (α_T,num_) using the San Diego (2016) mechanism. It can be observed that the percentage differences between the measurements and predictions ranged from 3 to 5%.

**Table 4:** Numerical and Experimental Results of Temperature Exponents Determined by Means of the Power Law Relation

Syngas mixtures	α_T,exp_	α_T,num_	Percentage difference (%)
S1	2.4048	2.2995	4.3
S2	2.1293	2.2389	5.1
S3	2.1212	2.1831	2.9

The increase in H_2_ concentration within the fuel mixture resulted in an increase of the temperature exponent, due to the higher thermal and mass diffusivity of H_2_ and H. For mixture S3 (9% H_2_ by volume), the exponent was 2.1212, while for mixture S1 (15% H_2_ by volume), it was 2.4048, reflecting a variation of 11%.

Varghese and Kumar^34^ determined the temperature exponent for a syngas mixture with the same composition as mixture S1, using the divergent channel method to measure the adiabatic laminar flame speed. The value found by the authors was 2.76. However, this temperature exponent was determined for an unburned mixture temperature between 520 and 635 K, while in the present work, the exponent was determined for *T*_u_ between 300 and 400 K. Furthermore, using this temperature exponent, the authors applied the power law relation and extrapolated *T*_u_ to 300 K, obtaining *S*_L0_ = 17 ± 1 cm/s. This value is about 16% (3.2 cm/s) lower than the adiabatic laminar flame speed measured in the present work (*S*_L0_ = 20.3 ± 1 cm/s). This difference may have occurred due to the wide range of *T*_u_ extrapolations applied by the authors or due to the experimental uncertainties in both works.

The second method applied to determine the temperature exponent was the activation energy method, which is presented in Section 3.1. In this method, the temperature exponent (α_Ea_) is determined by means of the relationship developed by Han et al.,^17^ which relates *E*_a_ to α_Ea_. Figure 5 presents the temperature exponent as a function of the unburned mixture temperature for the syngas mixtures S1, S2, and S3, with *P* = 0.1 MPa and Ø = 0.9. The temperature exponents were determined with the activation energy obtained experimentally, as shown in Figure 4, for *T*_u_ = 298 K.

**Figure 5:**
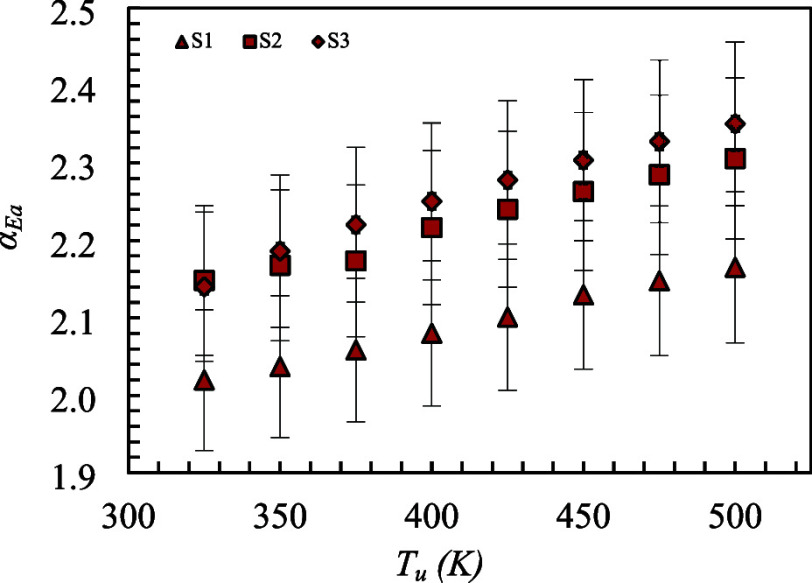
Experimental temperature exponent as a function of the unburned mixture temperature for mixtures S1, S2, and S3 (*P* = 0.1 MPa and Ø = 0.9).

An increase in the temperature of the unburned mixture was found to result in a corresponding rise in the temperature exponent. For mixture S2, within the *T*_u_ range of 325 to 400 K, the measured temperature exponent varied from 2.15 to 2.22. In comparison, the value obtained using the traditional method (Table 4) was 2.13, corresponding to a relative difference of approximately 1% to 4%. For mixture S3, over the same *T*_u_ range, the temperature exponent ranged from 2.14 to 2.25, indicating a deviation of about 1% to 6%. Mixture S1 exhibited the largest discrepancy when compared to the traditional method, with differences ranging from 13% to 16% within the 325–400 K interval.

The reduction of the H_2_ concentration in the syngas mixture (S1 to S3) resulted in an increase in the temperature exponent (α_Ea_) obtained experimentally. However, this trend could not be clearly identified due to the measurement uncertainties, as represented by the error bars.

Despite the differences in the values of the temperature exponents obtained by different methods, the effect of these variations on the estimation of the adiabatic laminar flame speed for different unburned mixture temperatures was not significant. Figure 6 shows the adiabatic laminar flame speed as a function of the unburned mixture temperature for mixtures S1, S2, and S3. The adiabatic laminar flame speed was extrapolated by means of the power-law equation, using the temperature exponents determined by the *E*_a_ method and the traditional method. The results were compared with the *S*_L0_ measured experimentally in the present work (symbols) and obtained numerically by means of the San Diego (2016) mechanism. All analyses were performed for Ø = 0.9 and *P* = 0.1 MPa.

**Figure 6:**
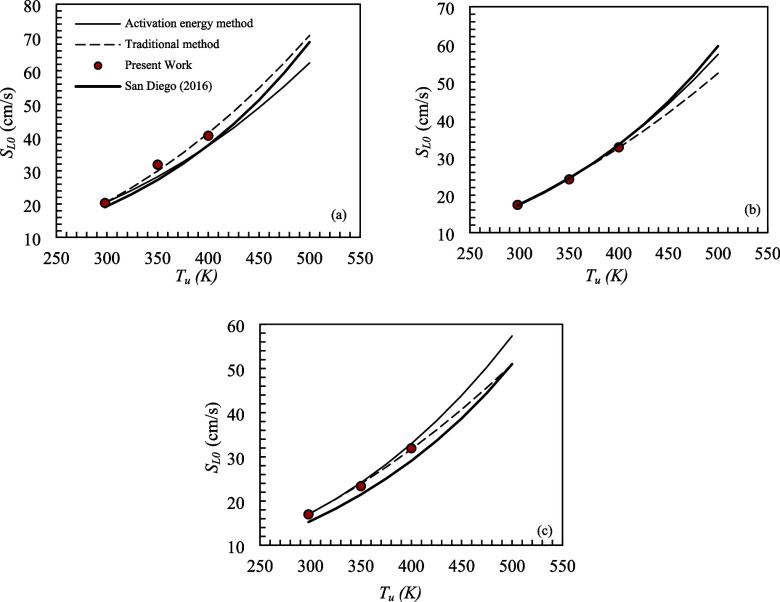
Estimation of the adiabatic laminar flame speed as a function of the unburned mixture temperature for mixtures (a) S1, (b) S2, and (c) S3, with Ø = 0.9 and *P* = 0.1 MPa.

The temperature exponent determined experimentally by means of *E*_a_ (α_Ea_) and used for *S*_L0_ extrapolation resulted in adiabatic laminar flame speeds with percentage differences in the order of 2 to 3% in relation to the values measured experimentally for mixtures S2 and S3. Only for mixture S1 was the percentage difference 7% and 11% (3 cm/s) for *T*_u_ equal to 400 and 350 K, respectively.

Comparing the adiabatic laminar flame speeds extrapolated using α_Ea_ with results obtained numerically, using the San Diego (2016) mechanism, for *T*_u_ between 298 and 500 K, it is noted that the percentage differences were on the order of 2 to 5% for S1, 1 to 2% for S2, and 12 to 13% for S3. However, differences of the same order of magnitude were observed in Section 3.2 when the experimentally measured *S*_L0_ values were compared with the numerical results obtained using the San Diego (2016) mechanism.

Regarding the temperature exponent determined experimentally using the traditional method (α_T_), it was observed that *S*_L0_ extrapolated presented percentage differences in relation to the experimental adiabatic laminar flame speeds in the order of 1 to 2% for mixtures S2 and S3; only for mixture S1 and *T*_u_ = 350 K, the difference was 6%. Comparing with the adiabatic laminar flame speeds determined numerically through the San Diego (2016) mechanism, the differences were on the order of 8 to 9% for mixture S1, 2 to 6% for mixture S2, and 5 to 10% for mixture S3, considering *T*_u_ between 298 and 500 K.

Furthermore, Varghese and Kumar^34^ measured the adiabatic laminar flame speed of a syngas mixture containing the same composition as fuel mixture S1. For Ø = 0.9, *P* = 0.1 MPa, and *T*_u_ = 450 K, the *S*_L0_ obtained by the authors was 47.5 cm/s, representing a percentage difference of 3 and 12% with respect to the *S*_L0_ estimated using α_Ea_ and α_*T*_, respectively.

## 4 Conclusions

The effect of the unburned mixture temperature on the adiabatic flame velocity and activation energy of multicomponent syngas mixtures was evaluated using the flat flame method with heat loss. Estimation of the activation energy enabled determination of the temperature exponent through the relationship between these parameters. This procedure was evaluated for mixtures of premixed methane and air. The results indicated that the temperature exponent obtained through *E*_a_ allows estimation of the adiabatic flame velocity for unburned mixture temperatures up to 100 K above the reference temperature, keeping the results close to experimental values reported in the literature.

Furthermore, increasing the unburned mixture temperature from 300 to 400 K had a more pronounced effect on mixture S1, characterized by the highest H_2_ concentration and an H_2_/CO ratio of 1, whereas the other tested mixtures exhibited lower H_2_/CO ratios of 0.43 and 0.67. Among the chemical kinetic mechanisms analyzed, the San Diego (2016) mechanism showed the greatest agreement with the experimental results, with differences between 13% and 1% for all conditions analyzed. A detailed sensitivity analysis of the reaction rate coefficients associated with each mechanism used in this study, in relation to the adiabatic laminar flame speed, is recommended for future work to elucidate the sources of discrepancies among the predicted results.

Regarding the activation energy of the syngas mixtures, increasing the unburned mixture temperature resulted in an increase in *E*_a_ for mixture S1, which has a higher H_2_ concentration. For mixtures S2 and S3, with H_2_ concentrations less than 15%, the activation energy remained approximately constant for unburned mixture temperatures between 300 and 400 K.

The temperature exponents of the syngas mixtures were evaluated by using both the activation energy and the traditional methods, ranging from 2.02 to 2.40 across all mixtures and methods. For mixture S1 with 15% H_2_, the difference in the determination of the temperature exponent between the methods was on the order of 13% to 16%. However, despite these differences, the results obtained in the adiabatic flame velocity estimation were very similar, particularly for unburned mixture temperatures differing by less than 100 K from the reference temperature.
